# Antioxidant Properties and Vasorelaxant Mechanism of Aqueous Extract *of Ricinodendron heudelotii* (Euphorbiaceae)

**DOI:** 10.1155/2024/3435974

**Published:** 2024-09-16

**Authors:** Jacquy Joyce Wanche Kojom, Calvin Zangueu Bogning, Edwige Laure Lappa, Christelle Stéphanie Sonfack, Augustine Nkojap Kuinze, Gisèle Etamé-Loé, Alain Bertrand Dongmo

**Affiliations:** ^1^ Department of Animal Biology and Physiology Faculty of Sciences University of Douala, PO Box 24157, Douala, Cameroon; ^2^ Department of Biological Sciences Faculty of Medicine and Pharmaceutical Science University of Douala, PO Box 2701, Douala, Cameroon

**Keywords:** endothelium, radical scavenging activities, vasodilatory effects

## Abstract

*Ricinodendron heudelotii* is a plant of the Euphorbiaceae family, used in traditional medicine to treat numerous diseases, including high blood pressure. The aim of this study is to evaluate the antioxidant and vasorelaxant effects of the aqueous extract of the stem bark of *R. heudelotii*.

The pharmacological studies were carried out using the aqueous extract obtained by infusion. The antioxidant capacity of *R. heudelotii* was assessed by in vitro tests with DPPH (2,2-diphenyl-1-picryl-hydrazyl), ABTS (2,2′-azino-bis (3-ethylbenz-thiazoline-6-sulfonic acid), iron-reducing capacity (FRAP), and inhibition of nitric oxide (NO) release. In vitro studies, the aortic rings obtained from adult Wistar albino rats of both sexes were used to determine the vasorelaxant effects of the extract of *R. heudelotii* on the NO and prostacyclin (PGI2) pathways as well as its involvement on various potassium channels were determined on intact or naked fragments of rat aorta precontracted with phenylephrine (10^−6^ M) or KCl (60 mM).

The aqueous extract of *R. heudelotii* exhibited a remarkable DPPH (EC_50_: 1.68 *μ*g/mL) and ABTS (EC_50_: 106.30 *μ*g/mL) and nitric oxide (53.71% inhibition at 1000 *μ*g/mL) radical scavenging activities as well as reducing power (absorbance of 1.56 at 1000 *μ*g/mL). The nitric oxide inhibitor, N_G_-nitro-L-arginine methyl ester (L-NAME), and prostacyclin inhibitor, indomethacin, significantly attenuated the vasodilatory effect of *R. heudelotii*. Tetraethylammonium could not inhibit the vasodilatory effect of the extract, unlike glibenclamide and barium chloride.

*Ricinodendron heudelotii* extract possesses antioxidant properties and vasorelaxing effect linked to endothelium-related factors, and this relaxation was partially mediated mainly through the inhibition of K_ir_ and K_ATP_ channels.

## 1. Introduction

Recently, many cardiovascular disorders are often treated with vasodilator drugs that act directly on the vascular smooth muscle, causing vasodilation, indirectly by stimulating the release of endogenous vasorelaxant factors or by inhibiting the release of vasoconstrictive factors [1, 2]. Preclinical studies and clinical trials have also indicated that antioxidant therapy is important for the management of hypertension, using antioxidant compounds such as alpha-tocopherol [3], ascorbic acid [4], and polyphenols with others [5]. Therefore, improving vasorelaxation and inhibiting oxidative stress are valuable strategies for fighting hypertension.

Effective synthetic drugs for the treatment of hypertension exist, but despite their efficacy, they have various adverse effects [6]. Nowadays, the utilization of traditional herbal plants as novel therapeutic agents plays a pivotal role in the management of cardiovascular diseases, especially hypertension [7]. In addition, natural substances which possess antioxidant and vasorelaxant properties have been the target of studies and are increasingly recognized for their use to prevent or treat hypertension [8].

Medicinal plants with antioxidant activity and rich phenolic compounds have been reported to have a number of biological activities [9–11]. In addition, natural substances which possess antioxidant and vasorelaxant properties have been fully exploited and are increasingly recognized for their use to prevent or treat hypertension [8].

*Ricinodendron heudelotii* (Baill.) Pierre (Euphorbiaceae) is an endemic species from tropical African rainforests [12] commonly called “Djansang” or “Essessang” in different areas in Cameroon. It is a large tree that grows throughout the humid lowland rainforest of Cameroon [13, 14]. In traditional medicine, different parts of the tree are used for the treatment of various diseases. The bark extract is used to cure cough, malaria, anemia, cancer, intestinal disease, dysentery, and as an antidote to poison [13, 15, 16]. It is also used as an aphrodisiac and diuretic [13, 17]. *Ricinodendron heudelotii* is well documented for some pharmacological properties, among which are antimicrobial, antioxidant, and anti-inflammatory activities [16, 17]. Its oleaginous nuts are harvested by the local population in Cameroon for both consumption and marketing [18].

Phytochemical studies revealed the presence of dinorterpenoids (heudelotenol; heudelotinone) as well as E-ferulic acid, octacosylate, and some natural chemopreventive agents [13, 19]. In our previous studies, the characterization of the phytochemicals carried out by HPLC-ESI-Q-TOF indicated the presence of a number of alkaloids and showed the antihypertensive activity of the aqueous extract of *R. heudelotii* [20]. Thus, in continuation of the pharmacological studies of this plant as a potential antihypertensive agent, the present study was, therefore, undertaken to assess the mechanism of action and antioxidant effects of the aqueous extract from the stem bark of *R. heudelotii*.

## 2. Materials and Methods

### 2.1. Drugs, Chemicals, and Reagents

All chemicals, drugs, and reagents used in this investigation were of analytical grade. N_G_-nitro-L-arginine methyl ester (L-NAME), indomethacin, dimethyl sulfoxide (DMSO), phenylephrine hydrochloride (PE), acetylcholine chloride (Ach), glibenclamide, tetraethylammonium (TEA), barium chloride (BaCl_2_), methanol, calcium chloride (CaCl_2_) ferric chloride (FeCl_3_), potassium ferricyanide (K_3_Fe(CN)_6_), Griess reagent, sodium nitroprusside (SNP), 2,2-diphenyl-1-picrylhydrazyl (DPPH), potassium persulfate (K_2_S_2_O_8_), 2,2-azino-bis(3-ethylbenzothiazoline-6-sulphonic acid) (ABTS), ascorbic acid (vitamin C), and 6-hydroxy-2,5,7,8-tetramethylchroman-2-carboxylic acid (Trolox) were obtained from Sigma Chemical (Germany).

All compounds were dissolved in distilled water, except indomethacin and glibenclamide that were dissolved in DMSO 5% and DPPH in methanol.

### 2.2. Collection of Plant Material, Identification, and Extraction

*Ricinodendron heudelotii* was collected in December 2017 at Malantouen, West Region-Cameroon by Dr Tacham, a botanist on duty at the University of Bamenda. The plant was identified at the National Herbarium in Yaounde (Cameroun) in comparison with the existing voucher's specimen, deposited under number 19695 SRF/Cam. The stem bark of *R. heudelotii* was cut out, dried in the shade, and then crushed. One hundred grams (100 g) of powder were infused in distilled water (1 L) preheated to 100°C for 20 min. After filtration through Whatman filter paper N°.3, the filtrate was evaporated at 40°C using an oven, yielding 1.21 g powder (w/w: 1.21%).

### 2.3. Antioxidant Assays

#### 2.3.1. DPPH Radical Scavenging Assay

The antioxidant activity of *R. heudelotii* against DPPH was determined according to the method described by Yan, Nagata, and Fa [21] with minor modifications. Briefly, a stock solution (5 mg/mL) of the crude extract was prepared in methanol. Serial dilutions were carried out to obtain concentrations of 1, 5, 10, 50, 100, 500, and 1000 *μ*g/mL. Diluted solutions (1 mL each) were mixed vigorously with a methanolic solution of DPPH (0.004%, 1 mL) and allowed to stand at room temperature for 30 min. Ascorbic acid was used as standard. The absorbance of the mixture was measured using a spectrophotometer at 515 nm. The absorbance of the DPPH radical with no antioxidant (blank) was also recorded. The experiment was done in triplicate and the mean absorbance was determined for each concentration. The DPPH radical scavenging activity (%) of the sample of ascorbic acid was calculated as follows [22]:

 $$\begin{array}{c} DPPH\;radical\;scavenging\;activity\left(\%\right)=\left(\frac{Ablank-Asample}{Ablank}\;\right)\times 100 \end{array}$$

where *A*blank is the absorbance mixture of DPPH work solution at *t* = 0 min and *A*sample is the absorbance of the mixture of sample/standard and DPPH work solution at *t* = 30 min.

#### 2.3.2. ABTS^•+^ Radical Cation Scavenging Assay

The antioxidant activity of *R. heudelotii* was evaluated by ABTS radical cation (ABTS•+) decolorization assay with slight modifications [23]. Briefly, the ABTS•+ solution was obtained by mixing 7 mM ABTS solution and 2.45 mM potassium persulfate in water, and the mixture was stored at room temperature for at least 16 h in the dark. The solution was then diluted with phosphate buffer (0.2 M, pH 7.4) to an absorbance of 0.750 ± 0.02 at 734 nm before use. After that, 100 *μ*L of each sample at different concentrations (1, 5, 10, 50, 100, 500, and 1000 *μ*g/mL) were mixed with 1000 *μ*L of ABTS radical cation working solution and after 20 min, the absorbance was read at 734 nm. An appropriate solvent blank (with no antioxidant) was run in each assay. All measurements were performed in triplicate. Trolox was used as a standard substance and the ABTS radical scavenging activity was calculated as follows [22]:

 $$\begin{array}{c} ABTS\;radical\;scavenging\;activity\left(\%\right)=\left(\frac{Ablank-Asample}{Ablank}\;\right)\times 100 \end{array}$$

where *A*blank is the absorbance of the mixture of distilled water and ABTS work solution and *A*sample is the absorbance of the mixture of sample/standard and ABTS work solution.

#### 2.3.3. Nitric Oxide (NO) Radical Scavenging Assay

The NO radical scavenging activity of *R. heudeloti* was determined according to the method of Marcocci et al. [24] with some modifications. Briefly, 450 *μ*L of extract at different concentrations (1, 5, 10, 50, 100, 500, and 1000 *μ*g/mL) were added to 50 *μ*L of sodium nitroprusside (10 mM prepared in 200 mM phosphate buffer, pH = 6.6) and incubated at 25°C for 3 h. The samples were then reacted by adding 250 *μ*L of Griess reagent (1% sulphanilamide+0.1% naphthylethylenediamine dihydrochloride in 2% phosphoric acid), and the absorbance was read at 540 nm. Each experiment was done in triplicate, ascorbic acid was used as a standard, and the NO radical scavenging activity was calculated as follows [22]:

 $$\begin{array}{c} NO\;radical\;scavenging\;activity\;\left(\%\right)=\left(\frac{Ablank-Asample}{Ablank}\;\right)\times 100 \end{array}$$

where *A*blank is the absorbance of the mixture of distilled water, the SNP solution, and the Griess reagent and *A*sample is the absorbance of the mixture of the sample/standard, the SNP solution, and the Griess reagent.

#### 2.3.4. Ferric Reducing Antioxidant Power Assay

The reducing power of *R. heudelotii* was estimated spectrophotometrically following the procedure of Benzie & Strain [25]. Briefly, 1000 *μ*L of each sample at different concentrations (1, 5, 10, 50, 100, 500, and 1000 *μ*g/mL) was mixed with 1000 *μ*L of potassium ferricyanide (1%, w/v) solubilized in phosphate buffer (pH 6.6, 20 mM) and incubated at 50°C for 20 min. Then, 1000 *μ*L of trichloroacetic acid (10%, w/v) was added followed by centrifugation at 3000 rpm for 10 min. The supernatant obtained (1000 *μ*L) was mixed with 1000 *μ*L of distilled water and 200 *μ*L of ferric chloride (0.1%, w/v). After incubation (30 min), the absorbance was measured at 700 nm against the blank. The blank contained all reagents except the extract and ascorbic acid was used as standard. All experiments were carried out in triplicate.

### 2.4. Vasorelaxant Assays of *R. heudelotii*

#### 2.4.1. Animal Conditions

Both adult male and female Wistar rats of 12–16 weeks, weighing 250–300 g were randomly selected from our local colony and raised in the animal house of the Faculty of Science, University of Douala, Cameroon. The rats were maintained at room temperature (12 h light/dark cycle, at 27°C) with free access to food and water. They were allowed to adapt to their environment for 2 weeks prior to the start of experiments. All procedures were approved by the Institutional Ethics Committee of the University of Douala (N°2040 CEI-UDo/06/2019/T) according to the guidelines established for the protection of animals used in experiments.

#### 2.4.2. Preparation of Isolated Rat Aortic Rings

The aorta rings were prepared as a method previously described by Dongmo et al. [26]. A total of 42 rats (7 groups, *n* = 6) were killed by cervical dislocation. The thoracic aorta was quickly and gently removed, cleaned of adherent connective tissue, and cut into rings (3–4 mm in length). Rings were gently introduced between two stainless steel hooks and placed in an organ chamber (Emka technologies, Paris) containing 20 mL of modified Krebs–Henseleit solution gassed with 95% O_2_ and 5% CO_2_ and maintained at 37°C and pH 7.4. One hook was connected to an isometric force transducer (Emka Technologies, USA), and the resting force of the samples after mounting on the hook was first set to 9.8 mN with a micromanipulator. The aortic rings were allowed to stabilize for 1 h with continuous changing of the bath solution until a constant base force was established. Data were continuously recorded with IOX data acquisition software from Emka Technologies. In some rings, the endothelium was removed by gently rubbing the intimal surface with a cotton swab. To check the functionality of the preparations, contractions of the aortic rings were elicited by adding 30 mM KCl. The bath solution was replaced until the resting tone was recovered [27].

#### 2.4.3. Experimental Procedure

For each experiment, six aortic rings derived from six rats were used. Before starting each experiment, the functional integrity of the endothelium was confirmed by evaluating the ability of acetylcholine (10^−6^ M) to produce relaxation in the aorta rings precontracted with phenylephrine (10^−6^ M). Relaxation of ≥ 60% indicated the presence of a functional or intact endothelial layer, while the lack of relaxation (≤ 10%) indicated the successful removal of the layer [28]. Following verification of endothelium integrity, the bath solution was renewed, and after the stabilization period, different experiment sets were performed.

##### 2.4.3.1. Effects of *R. heudelotii* Extract on Aortic Contraction Induced by PE or KCl

These experiments were made to verify the *R. heudelotii* extract-induced relaxation effect. The endothelium-denuded aortic rings were precontracted with PE (10^−6^ M) or KCL (60 mM). After the plateau was attained (contraction became stable), *R. heudelotii* was added cumulatively (1, 3, 10, 30, 100, 300, and 700 *μ*g/mL), and the vasorelaxant effect on the aortic rings was calculated as a percentage of contraction in response to PE or KCl [29].

##### 2.4.3.2. Effects of *R. heudelotii* in the Presence of L-NAME or Indomethacin

To determine the role of the endothelium in the vasorelaxant response of *R. heudelotii* extract, the aortic rings were pretreated with N-*ω*-nitro-L-arginine methyl ester (L-NAME, 10 *μ*M), a NO synthase inhibitor or indomethacin (10 *μ*M), a cyclooxygenase inhibitor for 20 min prior to precontraction induced by 1 *μ*M phenylephrine. The extract of *R. heudelotii* was then added cumulatively (1, 3, 10, 30, 100, 300, and 700 *μ*g/mL). After that, cumulative concentration-response curves of *R. heudelotii* extract were constructed and compared with the results obtained from aortic rings without inhibitors (control) [26].

##### 2.4.3.3. Effect of *R. heudelotii* in the Presence of Potassium Channel Blockers

To investigate the involvement of K^+^ channels, endothelium-denuded rings were incubated with TEA (10 mM), a nonselective K^+^ channel blocker, or BaCl_2_ (100 *μ*M), a K_ir_ channel blocker or glibenclamide (10 *μ*M), ATP-sensitive K^+^ (K_ATP_) channel blocker, for 20 min prior to contraction with phenylephrine (10^−6^ M). Then, the cumulative concentration–response curves of *R. heudelotii* were constructed and compared with those obtained with untreated rings (control, not treated with potassium channel blockers) [30].

### 2.5. Data Analysis

All data were expressed as means ± SEM. Analyses of concentration-response curves were performed by sigmoidal nonlinear regression (computer program: GraphPad Prism 5.00). Log values were used for the fitting. The resulting log EC_50_ values with their SEM values were converted to EC_50_ values and the corresponding SEM values. As a consequence, asymmetric errors result. The significance of differences was evaluated by means of one-way ANOVA (analysis of variance) followed by the Dunnett test. *p* values lower than 0.05 were considered to indicate significance.

## 3. Results

### 3.1. Antioxidant Activities of *R. heudelotii*

**Figure 1:**
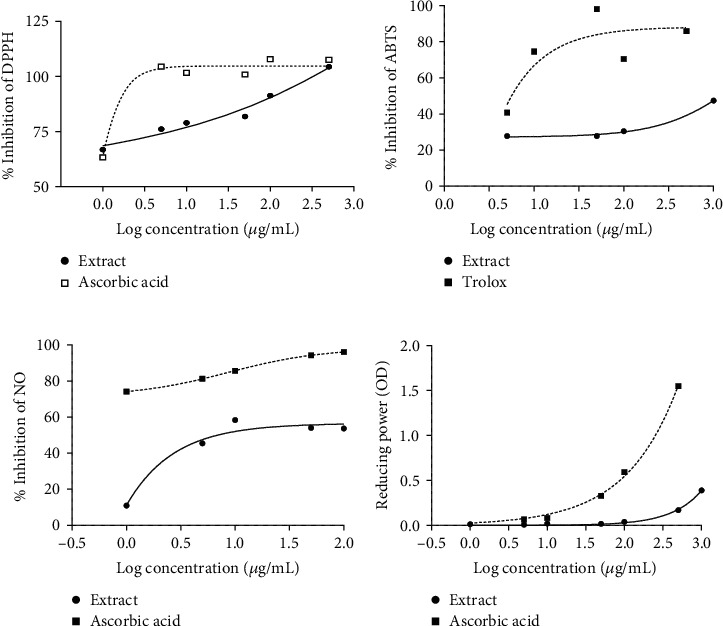
(a) DPPH radical scavenging activity, (b) ABTS radical cation scavenging activity, (c) NO radical scavenging activity, and (d) ferric reducing power activity of the aqueous stem bark extract of *Ricinodendron heudelotii*.

DPPH radical scavenging activity assay is depicted in Figure 1(a). The plant extract as well as ascorbic acid exhibited a strong radical scavenging activity against DPPH. The EC_50_ values of plant extract and ascorbic acid were 1.68 and 0.67 *μ*g/mL, respectively.

Furthermore, as shown in Figure 1(b), the stem bark extract of *R. heudelotii* exhibited ABTS radical scavenging activity at different concentrations, with a maximum value obtained at 1000 *μ*g/mL (the highest concentration tested). Even though the effect of the plant extract was 12 times less than that of Trolox used here as a standard antioxidant substance, theEC_50_value of the aqueous extract was 106.3 *μ*g/mL versus 8.47 *μ*g/mL for Trolox.

The extract of *R. heudelotii* showed a concentration-dependent scavenging activity against NO radicals (Figure 1(c)). The maximum activity was 53.71% at the highest concentration (1000 *μ*g/mL). The EC_50_ value of aqueous extract was 8.75 *μ*g/mL versus 4.69 *μ*g/mL for ascorbic acid.

Moreover, as shown in Figure 1(d), the aqueous extract of *R. heudelotii* exhibited a lower reducing power than ascorbic acid, while at the concentration of 1000 *μ*g/mL, its absorbance still reached 0.39 at 700 nm. At the highest concentration tested, the absorbance values were 1.56 and 0.16, respectively, for the aqueous extract of *R. heudelotii* and ascorbic acid (Figure 1(d)).

### 3.2. Vasorelaxant Effect of *R. heudelotii*

**Figure 2:**
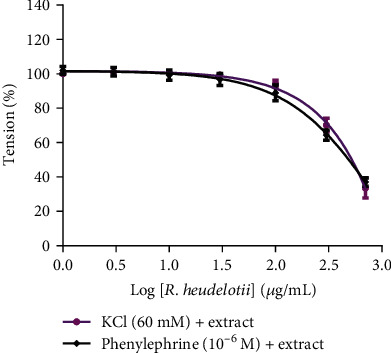
Concentration-dependent relaxant effects of *Ricinodendron heudelotii* on phenylephrine (PE, 10^−6^ M) or KCl (60 mM) precontracted rat aortic rings. The relaxant effects of *Ricinodendron heudelotii* were calculated as a percentage of contraction in response to PE or KCl. Values are mean ± S.E.M (*n* = 6).

Cumulative addition of *R. heudelotii* extract (1 to 700 *μ*g/mL) induced concentration-dependent relaxation in endothelium-denuded aortic rings precontracted by PE (10^−6^ M) or KCl (60 mM). The maximum relaxant effect (Emax) was 63.25% and 62.95% at the highest concentration (700 *μ*g/mL) tested, respectively (Figure 2).

**Figure 3:**
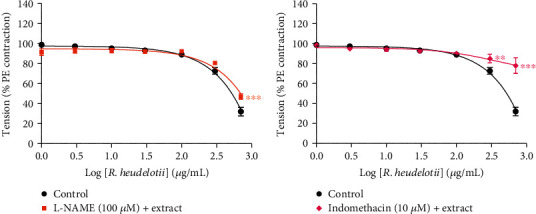
The effects of (a) L-NAME or (b) indomethacin on cumulative concentration responses of *Ricinodendron heudelotii* in intact aortic rings precontracted with phenylephrine (PE). The responses are expressed as % values (complete relaxation of PE-induced contraction = 100%). Values are mean ± SEM (*n* = 6). ^∗∗^*p* < 0.01 and ^∗∗∗^*p* < 0.001, significantly different in comparison with control.

As shown in Figures 3(a) and 3(b), pretreatment of intact aortic rings with L-NAME (100 *μ*M, a NO synthase inhibitor) or indomethacin (10 *μ*M, a cycloxygenase inhibitor) produced a significant change (*p* < 0.001) of the response, and vasorelaxant effect of *R. heudelotii* was markedly inhibited. In the presence of L-NAME or indomethacin, the Emax was 52.95% and 22.10%, respectively, versus 68.02% in the control aortic rings (absence of antagonists).

**Figure 4:**
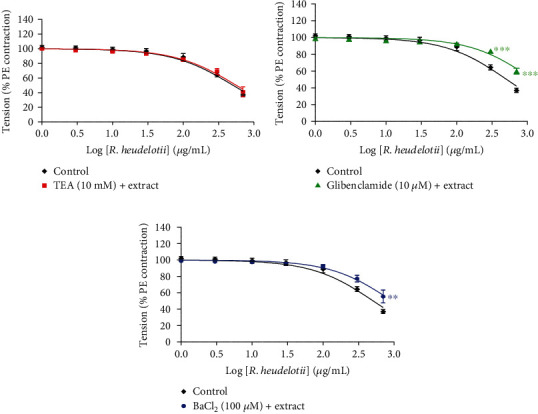
Relaxant responses induced by *Ricinodendron heudelotii* in rat aortic rings precontracted with phenylephrine (PE) in the absence or presence of (a) TEA, (b) glibenclamide, or (c) barium chloride. The responses are expressed as % values (complete relaxation of PE-induced contraction = 100%). Values are mean ± SEM (*n* = 6). ^∗∗^*p* < 0.01 and ^∗∗∗^*p* < 0.001, significantly different in comparison with control.

The vasorelaxant effect of the aqueous extract of *R. heudelotii* was markedly attenuated by preincubation with glibenclamide (10 *μ*M, a K_ATP_ blocker) or BaCl_2_ (100 *μ*M, a K_ir_ blocker). The Emax decreased from 62.95% in the absence of antagonists (control) to 40.15% and 44.46%, respectively, in the presence of glibenclamide and BaCl_2_ (Figures 4(b) and 4(c)). Pretreatment with TEA (10 mM) did not affect *R. heudelotii*-induced vasorelaxation (Figure 4(a)).

## 4. Discussion

Increasing evidence has indicated the key role of free radicals and reactive oxygen species (ROS) in the aetiology of degenerative pathologies such as Parkinson, Alzheimer, cancer, diabetes, and cardiovascular diseases [31, 32]. Both radicals and antioxidants are formed in normal cellular metabolism and in pathological conditions. Although the production of ROS is essential to health, when in excess, they can promote the oxidation of biological molecules, which leads to oxidative stress [33]. The importance of antioxidants lies in the fact that they are able to regulate the amount of these radicals in the body [34]. Thus, two antioxidant systems (enzymatic and nonenzymatic) are involved in the self-defence mechanism of the organism [35]. The sources of substances with antioxidant potential are various. Antioxidants present in the human diet may play an important role in disease prevention [36]. So, it is necessary to maintain the nonenzymatic system through the consumption of foods and plant substances rich in antioxidants [33] like *R. heudelotii* which is used in Benin and Cameroon in several food receipts [37]. In our previous study, it was reported that an extract of *R. heudelotii* stem bark possessed some indirect antioxidant activity since it interfered with the activities of some enzymes (SOD, CAT, and GSH) involved in the stress condition. Moreover, some compounds contained in this species have been shown as potential candidates for the development of new antihypertensive agents [38, 39].

Herein, we report the antioxidant effects of *R. heudelotii* extract using in vitro approaches and its vasorelaxant activity.

The antioxidant activities of the plant extract were assessed using in vitro methods such as DPPH, ABTS, NO radical scavenging assays, and reducing power tests. Among them are two colorimetric methods, DPPH and ABTS, which are conventionally used to determine the free radical scavenging activities of antioxidants present in a plant extract or synthetic compounds [40]. *Ricinodendron heudelotii* was found to exhibit potent free radical scavenging activity in both DPPH (EC_50_ = 1.68 *μ*g/mL) and ABTS (EC_50_ = 106.30 *μ*g/mL) assays. These results suggest that the extract of *R. heudelotii* would contain compounds that could act as free radical scavengers, that is, capable of donating hydrogen or electrons to a free radical in order to stabilize the odd electron which is responsible for radicals' reactivity [41]. The observed remarkable free radical scavenging activity of the extract may be explained by the presence of phenolic compounds such as dihydroxybenzoic acid, 3,4-dihydroxybenzaldehyde [42], and organic acids (citric acid) known for a good chelating ability towards metal ions [43]. Moreover, some alkaloids (magnoflorine) identified in this plant extract showed significant antioxidant activity as a DPPH-free radical scavenger and as against lipid peroxidation [44]. To confirm this hydrogen or electron-donating capacity, the reducing power of *R. heudelotii* was evaluated.

Reducing power is considered an important characteristic of antioxidants and is reflected in their ability to donate electrons. For this purpose, FRAP (Fe^3+^ →Fe^2+^) assay was performed [45]. The reducing capacity of this extract has been shown to be concentration-dependent, although lower than the activity of ascorbic acid (at 500 *μ*g/mL), suggesting that its antioxidant activity is at least partially due to its capacity to release electrons. Otherwise, several plant extracts have been reported to possess antioxidant activity exhibiting ferric reducing power in vitro [46]. The relationship between the contents of phenolics in the plant extract and its ability to reduce ferric ions has been reported [47].

NO is a free radical, its reduction or excessive production causes various affections [48]. From these results, the prooxidant activity exhibited by *R. heudelotii* as well as ascorbic acid on NO production suggest that this plant could be beneficial in the treatment of hypertension associated with endothelial dysfunction or an increase in NO bioavailability. Similar results on NO radical scavenging activity of polysaccharides from Snow Chrysanthemum (*Coreopsis tinctoria*) were obtained by Guo et al. [22]. Moreover, it has been also reported that seeds, fractions, and some compounds isolated in the leaves of *R. heudelotii* exhibited antioxidant properties *in vitro* using various tests [48, 49].

It is generally accepted that insufficient cellular protection against ROS contributes to vascular dysfunction and remodelling through oxidative stress. The most well-known is endothelium-dependent relaxation, which is impaired by a loss of NO activity in the vessel wall [50]. Therefore, the use of natural substances which possess antioxidant activity has gained considerable interest as protecting agents against vascular endothelial damage [8].

Our previous work has demonstrated the antioxidant and antihypertensive activities of the aqueous extract of the plant *in vivo* [20]. It is known that several mechanisms are involved in the antihypertensive activity of natural substances. In this study, the vasorelaxant activity of the plant extract and its possible mechanisms involved have been carried out.

The aqueous stem bark extract of *R. heudelotii* showed a concentration-dependent vasorelaxant effect on KCl as well as on PE-induced contraction. PE is an *α*-adrenergic agonist which induces vascular smooth muscle contraction via extracellular Ca^2+^ influx through receptor-operated channels and by internal calcium release from specific IP3 receptor (IP3R) channels in the sarcoplasmic reticulum membrane [51]. It is well established that extracellular Ca^2+^ influx through depolarization of the cell membrane and subsequent opening of VDCC is involved in KCl-induced contraction in vascular smooth muscle cells [52]. The stronger effect of *R. heudelotii* on PE than on KCl-induced contraction (622.7 *μ*g/mL vs. 508.9 *μ*g/mL) may indicate that inhibition of PE-induced intracellular Ca^2+^ release and of Ca^2+^ influx through receptor-operated channel play a more important role for the relaxing effect of *R. heudelotii* than inhibition of Ca^2+^ influx through L-type Ca^2+^ channels. The same results were obtained by Dongmo et al. [30] with tetra-acetylajugasterone, a new constituent isolated from *Vitex cienkowskii.*

The endothelium plays a crucial role in determining vasotone. The synthesis and release of vasorelaxing factors as well as vasoconstricting by the endothelium are involved in the regulation of vascular tone [30]. NO is one of the potent vasodilators secreted from the vascular endothelium which acts via the NO-cGMP pathway [53]. To evaluate the influence of vascular endothelium on the relaxing effect of the plant via activation of the NO-cGMP pathway, L-NAME, a blocker of NO synthesis, was used in experiments with rat aortic preparations. In this study, L-NAME affects the relaxant response induced by *R. heudelotii* only about 15% compared to control (without the antagonist), suggesting that another release of NO is not the only pathway involved in the vascular relaxation processes. Prostaglandins (PGs) such as prostacyclin constitute another group of EDRFs. Thus, indomethacin (a nonselective cyclooxygenase inhibitor) was used to evaluate the involvement of prostanoids in *R. heudelotii* induced vasodilatation. It was shown that indomethacin reduced significantly the effect of the extract of *R. heudelotii*-induced relaxation. It appears that indomethacin (a COX inhibitor) also affects the vasodilator response induced by the extract, suggesting that the production of prostanoids by the endothelial cells may be of some additional significance to the action of *R. heudelotii*. The same results were obtained by Dongmo et al. [26].

Furthermore, K^+^ channels also play an important role in the regulation of muscle contractility and vascular tension [54]. The activation of K^+^ channels causes hyperpolarization of the cell membrane leading to a reduction of the cytosolic Ca ^2+^ which induces vasorelaxation [55]. Diverse K^+^ channels are expressed in vascular smooth muscle, such as voltage-dependent K^+^ (Kv) channels, ATP-sensitive K^+^ (K_ATP_) channels, Ca^2+^-activated K^+^ (KCa) channels, and inward-rectifier K^+^ (Kir) channels [56]. To investigate potential K^+^ channel-related *R. heudelotii*-induced vasorelaxation, K^+^ channel blockers such as TEA (Kca blocker), glibenclamide (K_ATP_ blocker) and BaCl_2_ (K_ir_ blocker) were used. The vasorelaxant effect of *R. heudelotii* was significantly attenuated by glibenclamide and BaCl_2_ preincubation. These results suggest that the vasorelaxant effect of *R. heudelotii* is partially related to K_ATP_ and K_ir_ channels. However, TEA failed to inhibit the vasorelaxant effect of the aqueous extract of *R. heudelotii,* which clearly demonstrated that its action on K^+^ channels might not be mediated through K_Ca_ channels. In our previous study, the phytochemical analysis of the aqueous extract showed the presence of a number of alkaloid compounds (tetrahydropalmatine and magnoflorine), organic acids (citric acid, gluconic acid), and phenolic compounds (3,4-dihydroxybenzaldehyde) [20]. It has been reported that the relaxant activity of tetrahydropalmatine and mangnoflorine 3,4-dihydroxybenzaldehyde on rat aorta ring involved various mechanisms such as NO/cGMP signaling path-way, Ca^2+^ channels, and K^+^ channels rather than prostacyclin release [57–59].

Moreover, vasorelaxant effects of citric acid have also been described via an increase in NO production by the endothelium, but the other mechanisms are not clearly defined [60]. Aqueous extract of *R. heudelotii* has shown vasorelaxant activity using the same signaling pathways as these four compounds, except the prostanoids pathway. Thus, the vasorelaxant activity of the *R. heudelotii* extract observed could partly be due to the presence of some identified compounds such as tetrahydropalmatine, magnoflorine, citric acid, 3,4-dihydroxybenzaldehyde compounds known for their vasorelaxant property [57–60].

## 5. Conclusion

The present study indicated the interesting antioxidant activity of the stem bark extract from *R. heudelotii*. Moreover, its vasorelaxant properties observed are partially mediated via the NO pathway and K_ir_ and K_ATP_ calcium channel inhibition. These activities were probably related to the presence of the phenolic, alkaloid, and organic acid compounds in the extract. Thus, this plant represents a potential source of medicine for the treatment of cardiovascular diseases such as hypertension. Further studies will be necessary to elucidate other mechanisms of action and to characterize the active compounds responsible for the observed pharmacological effects.

## Data Availability

The data used to support the findings of this study can be obtained from the corresponding author upon request.
